# Supplementary material for: Layer‐Dependent Antiferromagnetic Chern and Axion Insulating States in UOTe
Source: Adv Sci (Weinh). 2026 Feb 19;13(20):e10247. doi: 10.1002/advs.202510247 (PMC13067864; doi:10.1002/advs.202510247)
Supplement: Supplementary file 1 — Supporting File: advs74205‐sup‐0001‐SuppMat.docx. [file ADVS-13-e10247-s001.docx]

*Supporting information of*
Layer-dependent antiferromagnetic Chern and axion insulating states in UOTe

Sougata Mardanya^1,*^, Barun Ghosh^2^, Mengke Liu^3^, Christopher Broyles^4^, Junyeong Ahn^5^, Kai Sun^6^, Jennifer E. Hoffman^3^, Sheng Ran^4^, Arun Bansil^7,8^, Su-Yang Xu^9,*^, Sugata Chowdhury^1,*^

^[[1]](#footnote-1)^ Department of Physics and Astronomy, Howard University, Washington, DC 20059, USA

^[[2]](#footnote-2)^ Department of Condensed Matter and Materials Physics, S. N. Bose National Centre for Basic Sciences, Kolkata 700106, India

^3^ Department of Physics, Harvard University, Cambridge, MA 02138, USA

^4^ Department of Physics, Washington University in St. Louis, St. Louis, MO 63130, USA

^5^ Department of Physics, The University of Texas at Austin, TX 78712, USA

^6^ Department of Physics, University of Michigan, Ann Arbor, MI 48109, USA

^7^ Department of Physics, Northeastern University, Boston, Massachusetts 02115, USA

^8^ Quantum Materials and Sensing Institute, Northeastern University, Burlington, MA 01803, US

^9^ Department of Chemistry and Chemical Biology, Harvard University, Cambridge, MA 02138, USA

**Table S1: Variation of bond lengths and interatomic distances with Hubbard U (in Å).**

| **Hubbard U (eV)** | **U–Te Bond length (Å)** | **U–O bond length (Å)** | **Interlayer U–U distance (Å)** | **U–U distance across VdW gap (Å)** |
| --- | --- | --- | --- | --- |
| 2 | 3.164 | 2.336 | 3.756 | 5.662 |
| 3 | 3.166 | 2.35 | 3.783 | 5.681 |
| 4 | 3.166 | 2.356 | 3.797 | 5.689 |
| 5 | 3.167 | 2.362 | 3.809 | 5.695 |
| 6 | 3.168 | 2.366 | 3.821 | 5.702 |
| 7 | 3.169 | 2.371 | 3.831 | 5.712 |

# Calculation of Magnetic exchange parameter


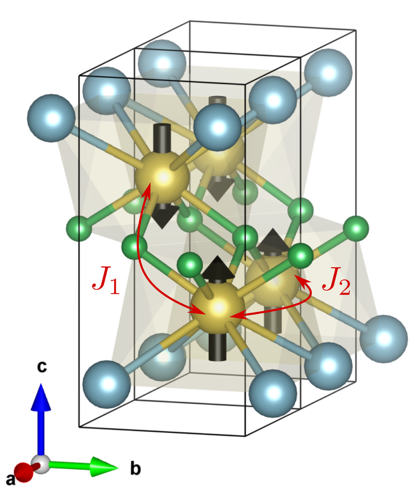
To determine the exchange parameter, we used the total energy difference method. We assumed that the low-energy magnetic interactions of UOTe can be described with the Heisenberg model $H=-\sum_{i\neq j} J_{ij}S_{i}.S_{j}$. We then performed total energy calculations for various magnetic configurations and solved for $J_{ij}$ by matching these energies with the model. For instance, to compute J1, we considered ferromagnetic and antiferromagnetic configurations, which have energies $E_{FM}=-JS^{2}$ and $E_{AFM}=JS^{2}$ respectively. Consequently, the magnetic exchange parameter is $J=\left( E_{AFM}-E_{FM} \right)/\left( 2S^{2} \right)$. Here, S is the spin magnitude, and for UOTe, the spin magnitude of $4^{+}$ is 1.

***Figure S1.*** *Magnetic exchange interactions in UOTe*


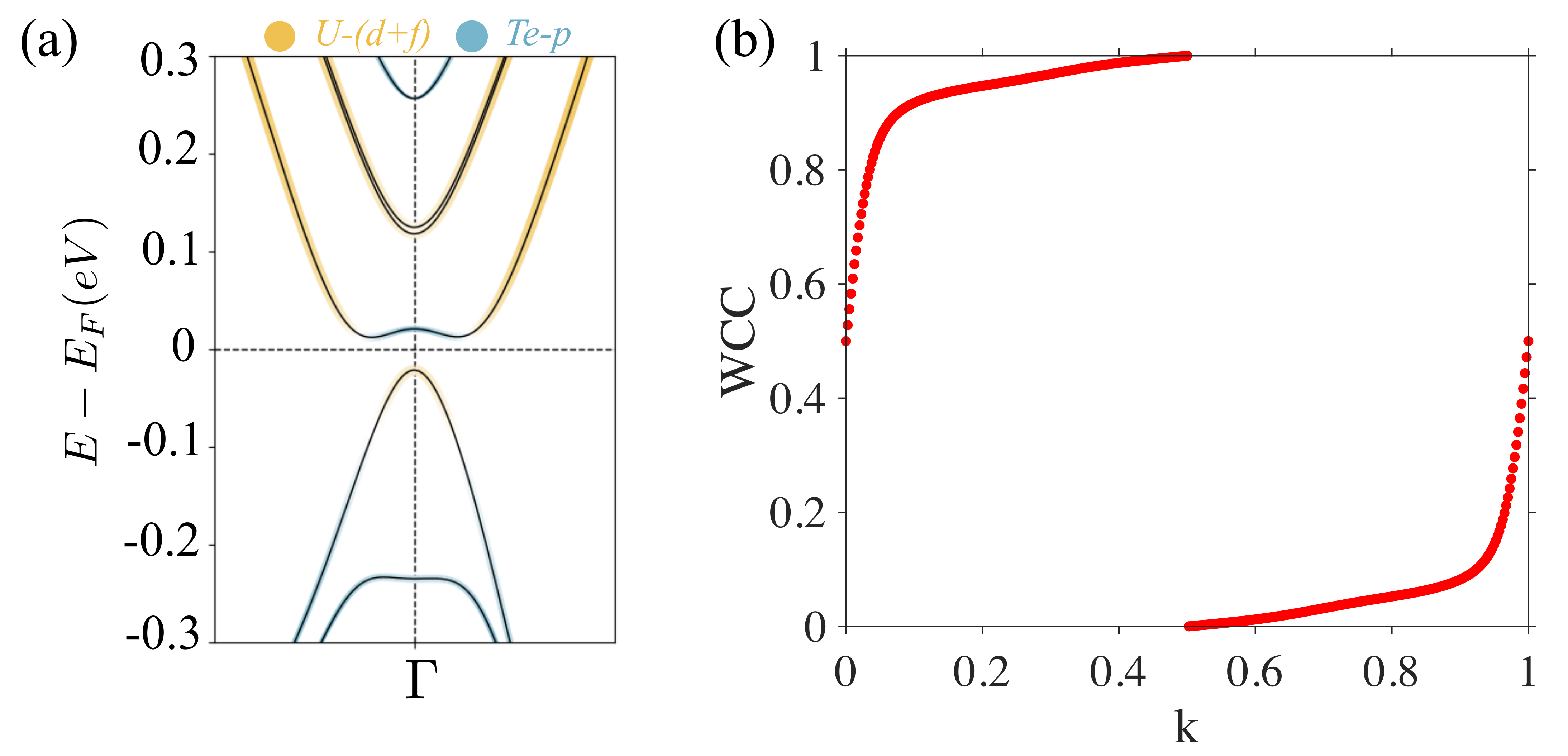


**Figure S2.** (a) The orbital resolved electronic structure of 2-layer UOTe. The yellow and blue colors represent the contribution from U-(d+f) and Te-p, which highlight the band inversion at the Γ point across Fermi energy. (b) Evolution of Wannier charge center winding the Brillouin zone one single time, indicating the C=1 Chern insulating phase.


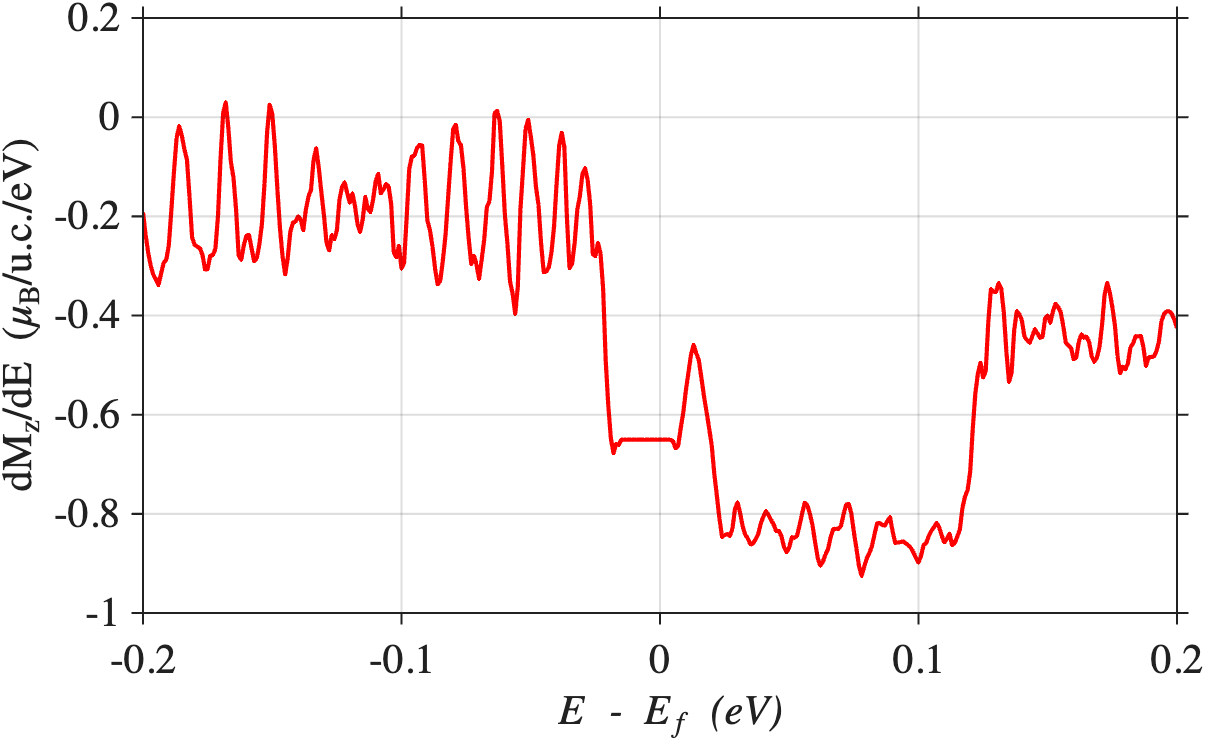


**Figure S3.** The variation of $dM/dE$ with chemical potential demonstrates a plateau-like feature within the bulk gap, confirming the topological nature of the orbital magnetization arising from the itinerant motion of electrons at the boundary.


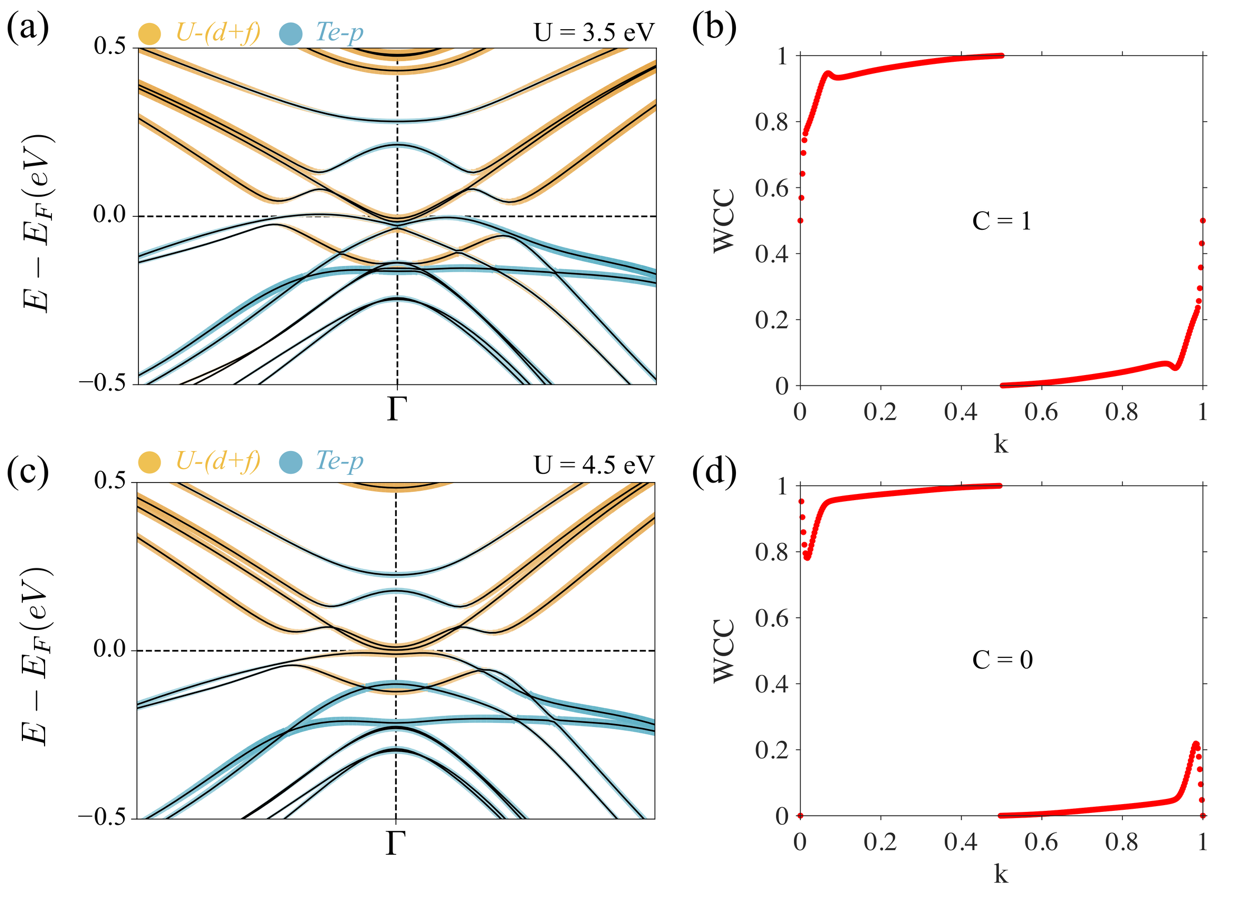
**Figure S4.** Orbital resolved band structure of 2-Layer UOTe for Hubbard U potential (a) U=3.5 eV and (b) U=4.5 eV. (c) and (d) represent the evolution of the Wannier charge center for the case of (a) and (b) to identify the topological character as C=1 and C=0, respectively.


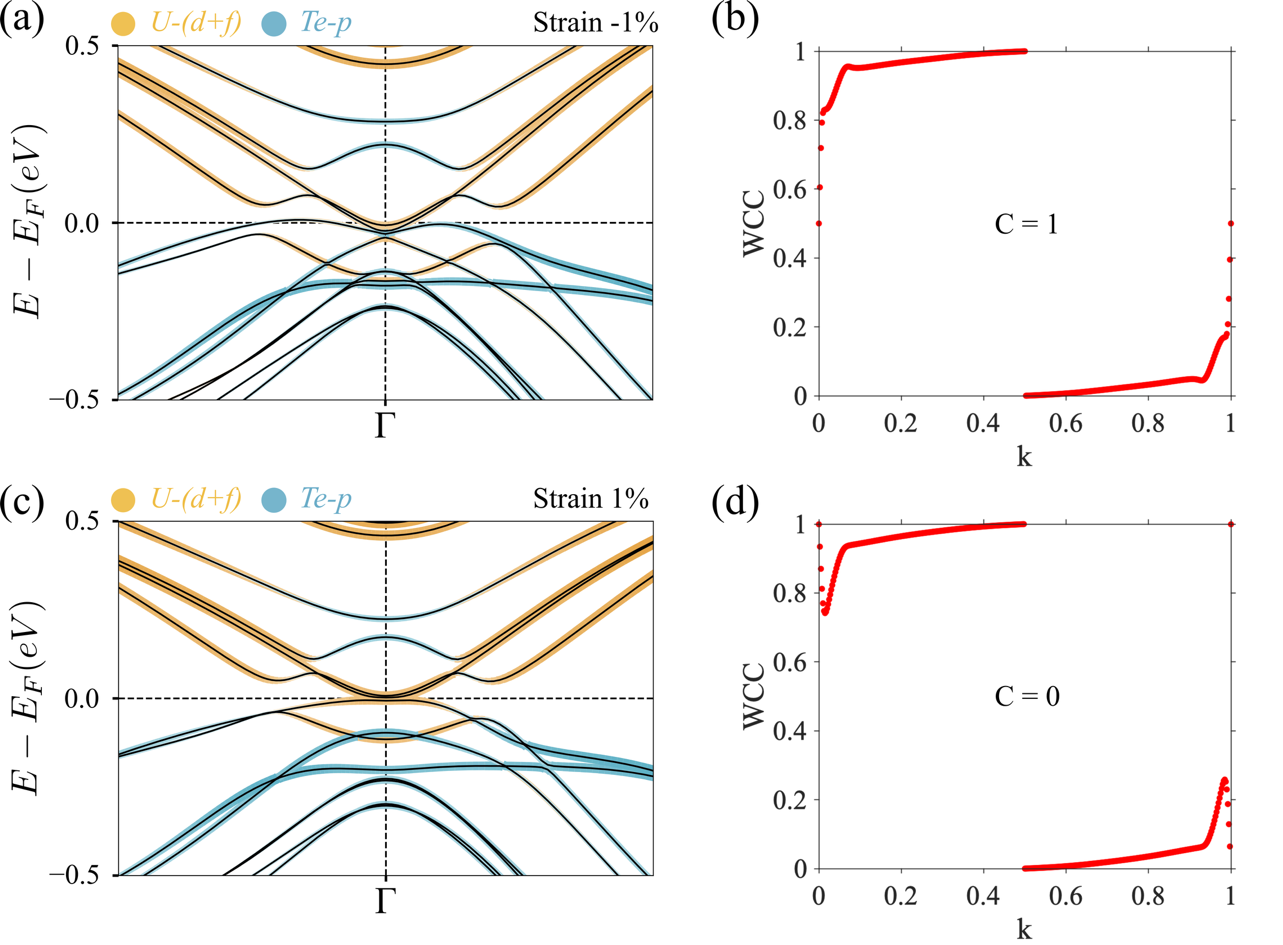


**Figure S5.** Orbital resolved band structure of 2-Layer UOTe for strain values at (a) -1% and (b) 1%. (c) and (d) represent the evolution of the Wannier charge center for the case of (a) and (b) to identify the topological character as C=1 and C=0, respectively.


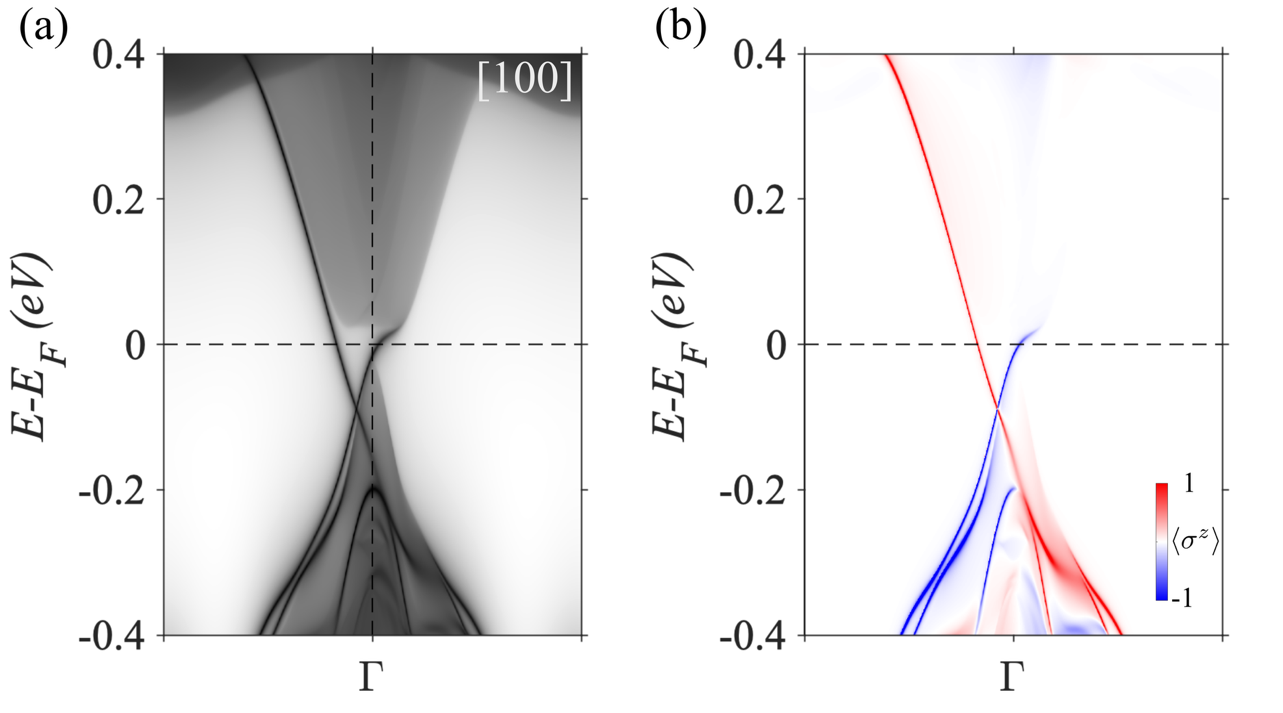


**Figure S6.** (a) Edge state spectrum of 3-Layer UOTe along the [100] direction showing the opposite moving chiral state. (b) The $\langle\sigma^{z}\rangle$ weighted edge spectrum shows fully spin-polarized edge states.

1. * Corresponding author [↑](#footnote-ref-1)
2. [↑](#footnote-ref-2)
